# Dual transcriptomics of virus-host interactions: comparing two Pacific oyster families presenting contrasted susceptibility to ostreid herpesvirus 1

**DOI:** 10.1186/1471-2164-15-580

**Published:** 2014-07-09

**Authors:** Amélie Segarra, Florian Mauduit, Nicole Faury, Suzanne Trancart, Lionel Dégremont, Delphine Tourbiez, Philippe Haffner, Valérie Barbosa-Solomieu, Jean-François Pépin, Marie-Agnès Travers, Tristan Renault

**Affiliations:** Ifremer (Institut Français de Recherche pour l’Exploitation de la Mer), Unité Santé Génétique et Microbiologie des Mollusques (SG2M), Laboratoire de Génétique et Pathologie des Mollusques Marins (LGPMM), Avenue de Mus de Loup, 17390 La Tremblade, France; Ifremer, Laboratoire Environnement Ressources des Pertuis Charentais (LERPC), Avenue de Mus de Loup, 17390 La Tremblade, France

**Keywords:** Interactions, Ostreid herpesvirus 1, Gene expression, Susceptibility, *Crassostrea gigas*

## Abstract

**Background:**

Massive mortality outbreaks affecting Pacific oyster (*Crassostrea gigas*) spat in various countries have been associated with the detection of a herpesvirus called ostreid herpesvirus type 1 (OsHV-1). However, few studies have been performed to understand and follow viral gene expression, as it has been done in vertebrate herpesviruses. In this work, experimental infection trials of *C. gigas* spat with OsHV-1 were conducted in order to test the susceptibility of several bi-parental oyster families to this virus and to analyze host-pathogen interactions using *in vivo* transcriptomic approaches.

**Results:**

The divergent response of these oyster families in terms of mortality confirmed that susceptibility to OsHV-1 infection has a significant genetic component. Two families with contrasted survival rates were selected. A total of 39 viral genes and five host genes were monitored by real-time PCR. Initial results provided information on (i) the virus cycle of OsHV-1 based on the kinetics of viral DNA replication and transcription and (ii) host defense mechanisms against the virus.

**Conclusions:**

In the two selected families, the detected amounts of viral DNA and RNA were significantly different. This result suggests that Pacific oysters are genetically diverse in terms of their susceptibility to OsHV-1 infection. This contrasted susceptibility was associated with dissimilar host gene expression profiles. Moreover, the present study showed a positive correlation between viral DNA amounts and the level of expression of selected oyster genes.

## Background

Ostreid herpesvirus type 1 (OsHV-1), the causative agent of major economic losses in the Pacific oyster industry, is a member of the family *Malacoherpesviridae* from the order *Herpesvirales* [1, 2]. It was in particular identified as the main cause of the massive mortality outbreaks of *C. gigas* spat recently reported in Europe, Australia and New Zealand [3, 4].

Within this context, the control of OsHV-1 infection is considered as a key element to maintain the competitiveness and to increase the sustainability of oyster industry. However, Pacific oysters as well as all other marine molluscs have unique features in terms of health management [5]. Not only do they live in open marine waters, directly exposed to pathogen infection but they also lack an adaptive immune system. Vaccination is therefore not an option to protect them against virus infection. As a consequence, most strategies currently used for other farmed animal species (e.g., cattle, fish) cannot be directly applied to Pacific oysters. One of the few applicable and promising approaches to limit the harmful effect of pathogens in oyster production is, selective breeding [6].

In animal populations, susceptibility to virus infection and the modulation of disease progression display inter-individual variability [7–9]. The study of host factors that control disease susceptibility relies on the multi-approach analysis of individuals demonstrating a differential response to OsHV-1 exposure/infection. This type of data will facilitate the identification of new targets with the potential to be integrated in innovative selection strategies. Genes that confer resistance to OsHV-1 may indeed be revealed by combining the “top-down” QTL approach to identify target genomic regions, with “bottom-up” transcript profiling. A QTL analysis for OsHV-1 DNA detection and quantification that was previously performed on five F_2_ full-sib families highlighted the relevance of further studying their respective genetic controls [10, 11].

Oyster gene expression has already been studied [12–14], particularly since the oyster genome was sequenced [15]. However, most studies relied on the use of oysters collected in the field which are subjected to multiple stressors and pathogens [12, 13, 16, 17]. In addition, only three studies reported OsHV-1 gene expression while the OsHV-1 cycle has never been described at a molecular level [18–20].

The aim of our study was to determine the genetic basis of differential susceptibility to virus infection among Pacific oysters. To overcome the variability of environmental factors, we reproduced the viral infection under controlled laboratory conditions. Sixteen Pacific oyster families were initially challenged with the virulent variant of OsHV-1, μVar [21]. Survival rates were evaluated five days post-injection and used as a measure of their susceptibility to OsHV-1. In a second step, two Pacific oyster families with contrasted susceptibility were selected for transcript profiling of 39 virus and five oyster immune related genes.

## Results

### Screening Pacific oyster families for OsHV-1 susceptibility

**Figure 1:**
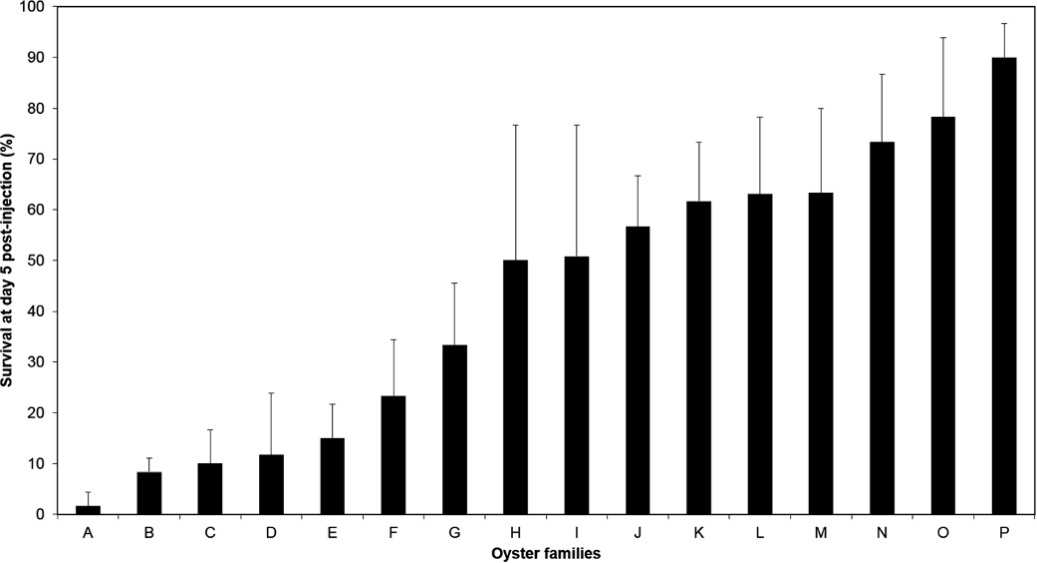
**Survival of 16 families of** ***Crassostrea gigas*** **oysters at 120 hours post infection during OsHV-1 challenges.** Two assays were conducted in triplicates for each family (n = 60). For all 16 families, 100% of survival was reported among negative controls injected with 100 μL of artificial seawater.

Two independent experimental infection trials were performed to strengthen the results in terms of survival rates of the 16 bi-parental families of Pacific oysters that were challenged. After infection, mortality was monitored daily for a period of 120 hours. Control batches systematically displayed 100% survival rates. Results showed that susceptibility to OsHV-1 was different among the tested oyster families (Figure 1). Survival rates were 2 ± 1.6% for family “A” and 90 ± 4.5% for family “P” 120 hours post infection (hpi) (Table 1). Based on their survival rates and Wilcoxon test (p > 0.05) (Table 1), families A and P were selected to represent the most extreme performances in terms of susceptibility to OsHV-1. A typifies the extremely sensitive family and P the less sensitive family with regard to infection by OsHV-1 (Figure 1).

**Table 1:** **Survival rates of the challenged families and identification of groups of families with significantly distinct performances**

Families	Wilcoxon test (p < 0.05)	Survival post infection (%)					
		0 hpi	24 hpi	48 hpi	72 hpi	96 hpi	120 hpi
A	a	100	100	32 (±9.5)	15 (±5)	3 (±3.3)	2 (±1.6)
B	ab	100	100	53 (±8)	25 (±4.3)	8 (±1.6)	8 (±1.6)
C	bc	100	100	47 (±7.1)	12 (±4)	10 (±3.6)	10 (±3.6)
D	bc	1oo	100	78 (±9.8)	25 (±7.6)	15 (±6.1)	12 (±6.5)
E	bc	100	100	47 (±5.6)	28 (±6.5)	17 (±3.3)	15 (±3.4)
F	bc	100	100	97 (±5.4)	75 (±11.4)	57 (±11.9)	33 (±6.5)
G	c	100	100	67 (±7.1)	47 (±9.5)	37 (±11.1)	33 (±8)
H	cd	100	100	92 (±5.4)	80 (±7.3)	60 (±12.1)	50 (±13.2)
I	def	100	100	100	90 (±3.3)	80 (±4)	70 (±4.9)
J	de	100	100	88 (±6.3)	66 (±8.5)	62 (±8.9)	60 (±7.9)
K	def	100	100	100	78 (±4.9)	72 (±7)	65 (±6)
L	de	100	100	90 (±6.3)	67 (±10.5)	62 (±10.6)	57 (±8.8)
M	ef	100	100	100	70 (±4.5)	63 (±4.8)	52 (±8.7)
N	ef	100	100	88 (±3)	80 (±5.8)	75 (±7.6)	73 (±6.7)
O	df	100	100	100	90 (±3.6)	73 (±8.7)	73 (±8.7)
P	g	100	100	100	95 (±5)	92 (±4.8)	90 (±4.5)

Groups based on the daily monitoring of mortality during 120 hours post infection (Wilcoxon test). Lowercase letters design a family group significantly different from all the others. All families were tested by pairs and several groups were identified.

### Comparing two families with contrasted susceptibility to OsHV-1 infection

**Figure 2:**
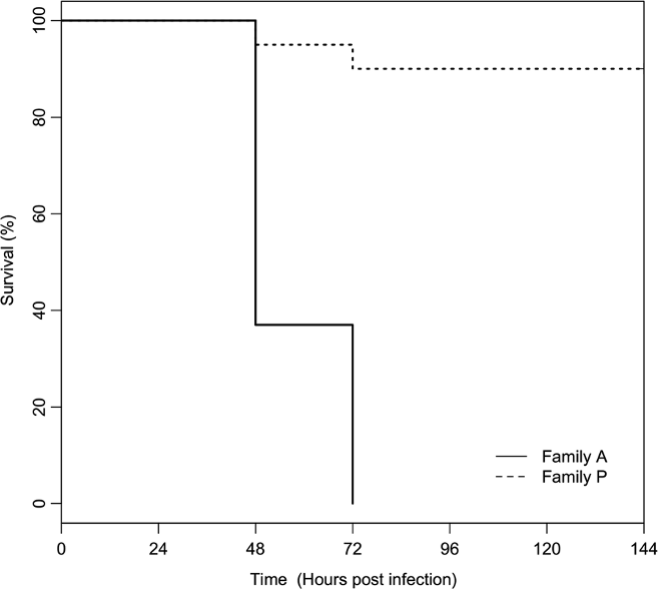
**Kaplan-Meier survival curves of two families of** ***Crassostrea gigas*** **during infection with OsHV-1.** For the third experiment, one tank was used (n = 20). For the two families, 100% of survival was reported among negative controls injected with 100 μL of artificial seawater.

The respective mean survival rates of families A and P were 37% and 95% at 48 hpi and 0% and 90% at 72 hpi (Kaplan-Meier, Figure 2). At later time points, family A was entirely dead whereas the survival rate was stable (89.5% 72 h and 144 hpi) for family P (Figure 2). As indicated previously, the survival rate was 100% for oyster controls.

**Figure 3:**
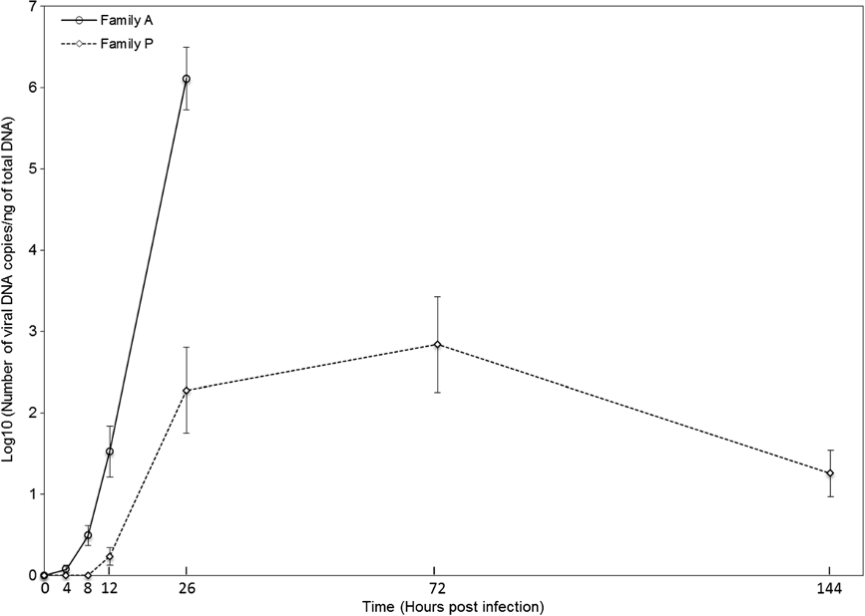
**Virus DNA detection curves by real time quantitative PCR in oyster from families A and P after injection OsHV-1 (average n = 12).** In both families, a result of 0 DNA copies/ng of total DNA was reported for negative controls injected with 100 μL of artificial seawater. Error bars represent ± standard deviation.

Virus DNA was initially detected at 4 hpi for family A and 12 hpi for family P (Figure 3). The mean amounts of viral DNA increased gradually during the course of infection in family A until all oysters died, whereas it increased up to 72 hpi and decreased until 144 hpi in family P (Figure 3). Viral DNA amounts were overall lower in family P than in family A (Figure 3). In family A, individual amounts of OsHV-1 DNA ranged from 0 to 3.2 × 10°, 0 to 1.7 × 10^1^, 0 to 2.8 × 10^3^, and 1.2 × 10^1^ to 9.5 × 10^6^ viral DNA copies per ng of total DNA at 4 hpi, 8 hpi, 12 hpi and 26 hpi, respectively. In family P, individual amounts of OsHV-1 DNA ranged from 0 to 1.4 × 10^1^, 0 to 1.3 × 10^5^, 0 to 9.1 × 10^5^ and 0 to 7.2 × 10^2^ viral DNA copies per ng of total DNA at 12 hpi, 26 hpi, 72 hpi and 144 hpi, respectively. No virus DNA was detected in oysters injected with artificial seawater.

### Detection and expression of OsHV-1 genes in A and P families

**Figure 4:**
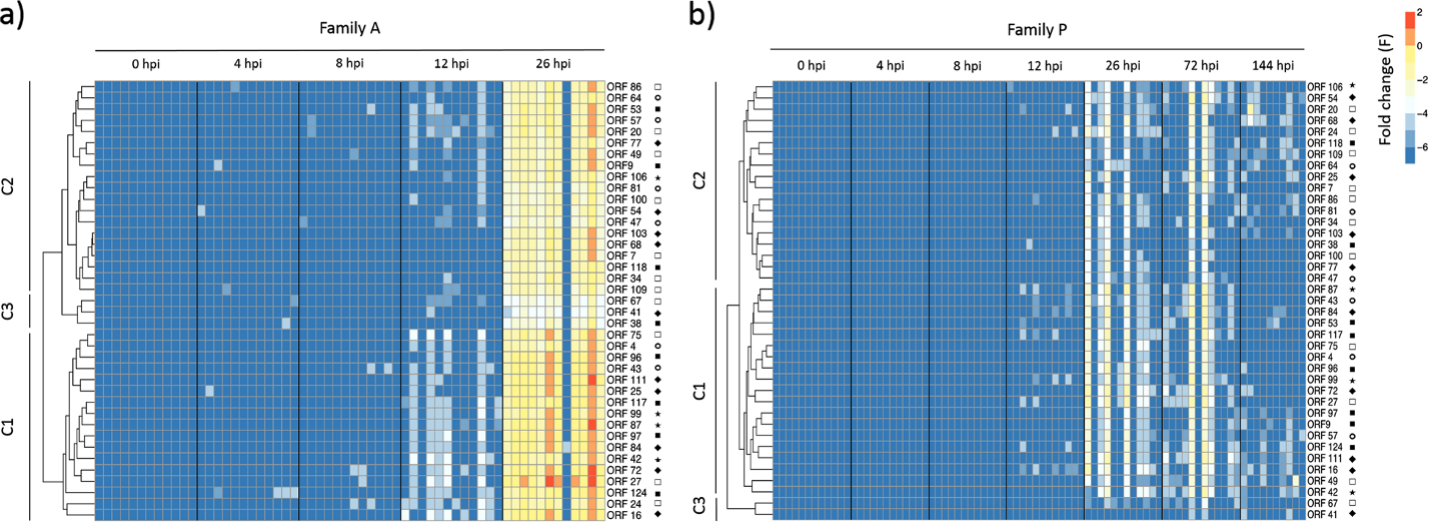
**Heatmap illustrating the level of expression of 39 OsHV-1 genes at different post infection time points in (a) family A and (b) family P.** Colors represent the fold change. Blue color (-7) was used to represent the lack of virus detection, whereas red color (+2) was employed for highly expressed genes. Each row corresponds to one of 39 viral genes and each column represents an individual (n = 12 per time). The five groups/families of genes were: Ο: putative protein; □: enzyme; ■ RING finger protein; **★**: Inhibitor apoptosis and ♦: membrane protein. The full list of genes is available in Segarra et al. [18]. Three clusters were generated for each family: C1, C2 and C3.

**Figure 5:**
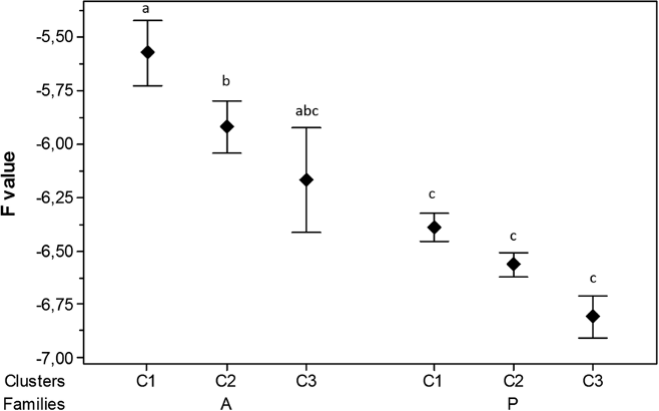
**Means of viral gene expression by clusters for each family.** Different lowercase letters indicate significant differences (Kruskal-Wallis and Dunn’s tests, p < 0.05) between clusters. Error bars represent ± standard deviation.

The level of expression in mantle tissue of 39 OsHV-1 genes was studied in both families (A and P) by real-time PCR. These particular genes were selected based on literature data and/or their specific function [1, 13]. The full list of OsHV-1 genes is available in Segarra et al. [18]. Normalized expression levels were calculated for each individual at each time point in both families. Two heatmaps were generated (Figure 4a and b). The most striking finding was the existence of different levels of gene expression between both families. All 39 ORFs were significantly over-expressed (Wilcoxon test, p < 0.05) in family A in comparison to family P, particularly at 26 hpi (F = 1.5 and F = -1, respectively). Moreover, initial detection of virus transcripts was performed as early as 4 hpi in family A but did not occur until 12 hpi in family P. In family P, expression levels peaked at 26 and 72 hpi before and decreased at 144 hpi. Differences in terms of expression levels were observed between individuals belonging to the same family and collected at identical time points. No viral transcript was detected at 0 hpi in either family.Virus transcripts were grouped by the euclidean method to produce a dendrogram comparing the expression levels of all 39 ORFs (Figure 4a and b). Three clusters were observed for each family. Cluster 1 corresponds to high expression levels, regrouped 17 genes in family A and 19 genes in family P (Figure 4a and b). Cluster 2, with medium expression levels, comprised 19 genes in family A and 18 genes in family P, (Figure 4a and b). The third and last cluster included three genes in family A and two genes in family P and was characterized by low expression levels (Figure 4a and b). Significant differences were observed between the three clusters (C1, C2 and C3) in family A whereas no difference was detected in family P for these clusters (Kruskal-Wallis and Dunn’s tests, p < 0.05) (Figure 5). However, 82% of the analyzed ORFs (32/39) were classified in the same cluster in both families: 15 genes belonged to cluster 1 (ORF 4, ORF 16, ORF 27, ORF 42, ORF 43, ORF 72, ORF 75, ORF 81, ORF 87, ORF 96, ORF 97, ORF 99, ORF 111, ORF 117, ORF 124); 15 genes to cluster 2 (ORF 7, ORF 20, ORF 34, ORF 47, ORF 54, ORF 64, ORF 68, ORF 77, ORF 81, ORF 86, ORF 100, ORF 103, ORF 106, ORF 109, ORF 118) and two genes to cluster 3 (ORF 41, ORF 67). In both families, three viral inhibitor of apoptosis (IAP) genes (ORF 42, ORF 87 and ORF 99) out of the four IPAs that were analysed pertained to cluster 1 (Figure 4a and b).

### Relative quantification of host gene expression in A and P families

**Figure 6:**
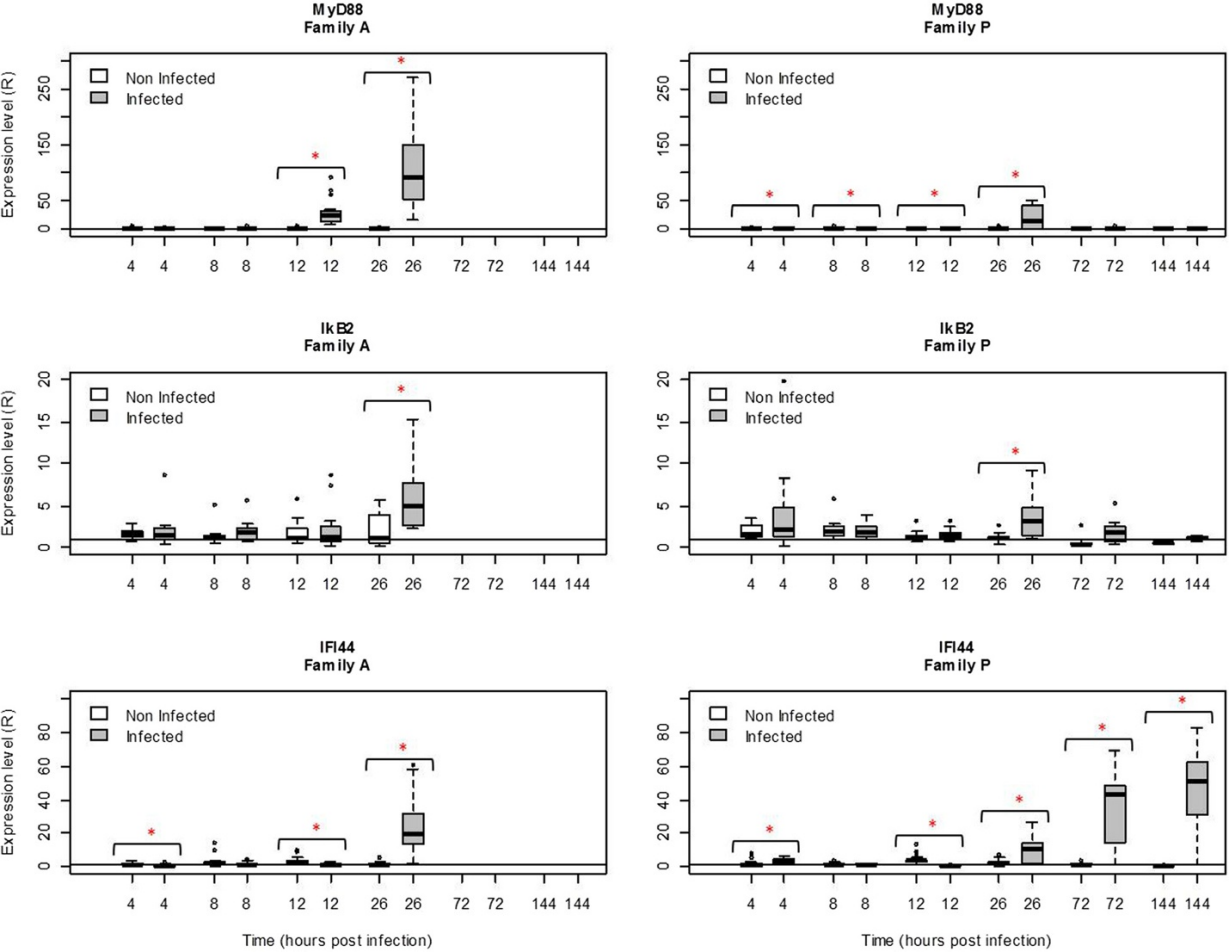
**Relative expression of three selected genes (MyD88, IkB2, and IFI44) in family A and family P, determined by real time PCR.** MyD88: myeloid differentiation factor 88, IkB2: Inhibitor of nuclear factor kappaB kinase beta, and IFI44: Interferon induced protein 44. Results at 4, 8, 12, 26, 72 and 144 hours post-infection. Expression levels were normalized to EF (mean ± SD, *n* = 12). A value = 1 is arbitrarily assigned to controls. *Significant difference of gene expression compared to controls by Mann–Whitney test (p < 0.05).

**Figure 7:**
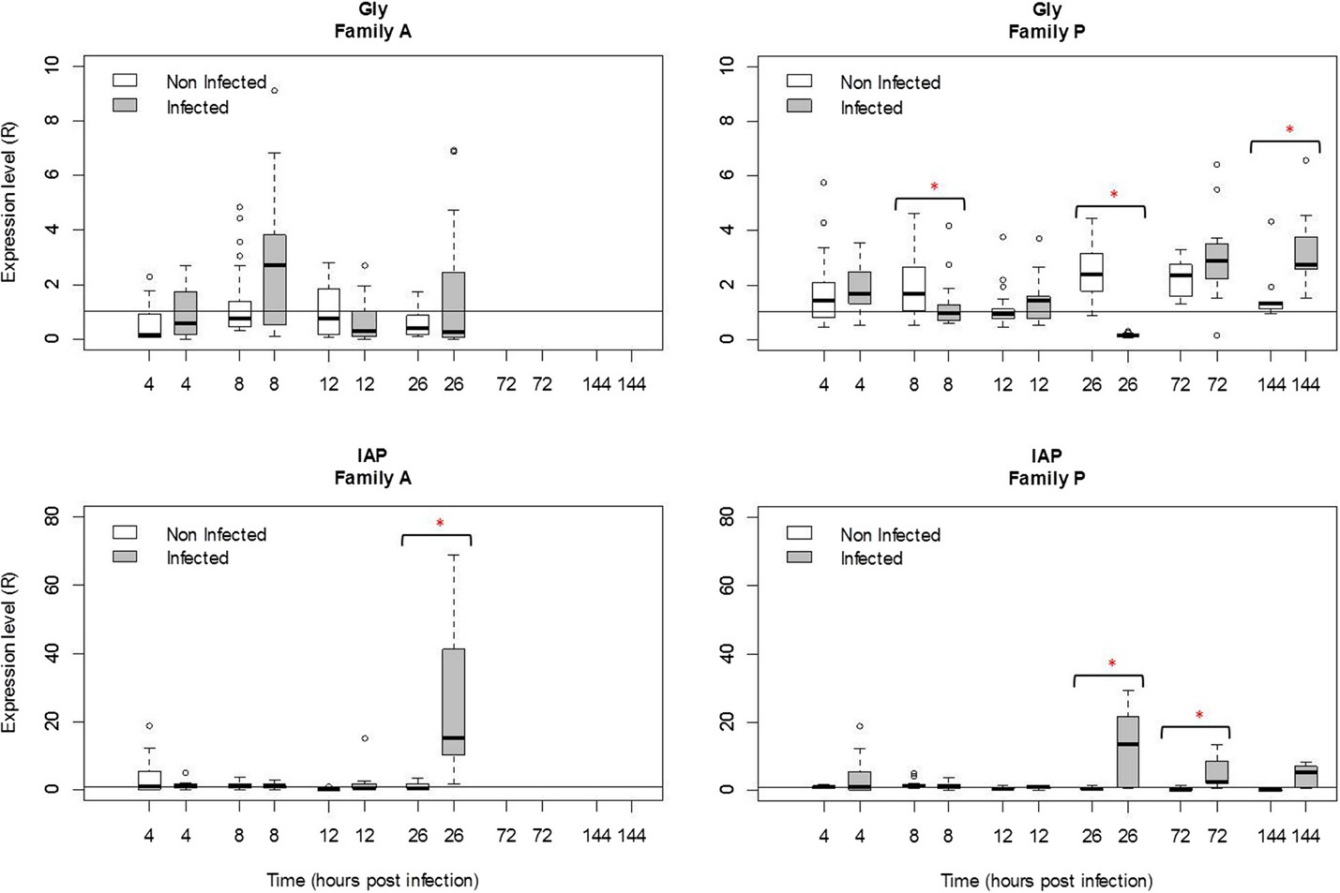
**Relative expression of two selected genes (Gly and IAP) in family A and family P, determined by real time PCR.** Gly: Glypican and IAP: Inhibitor of apoptosis. Results at 4, 8, 12, 26, 72 and 144 hours post-infection. Expression levels were normalized to EF (mean ± SD, *n* = 12). A value = 1 is arbitrarily assigned to controls. *Significant difference of gene expression compared to controls by Mann–Whitney test (p < 0.05).

In the third experiment, a significant increase or decrease in mRNA levels was reported for IFI44, MyD88, IAP and IkB2 at several times post injection in challenged oysters from both families as compared with negative controls (Figures 6 and 7). In family A, IFI44 gene expression was up-regulated at 26 hpi in infected oysters (R = 24.1) compared to control oysters (R = 1.3) (p < 0.05). In family P, IFI44 transcript level (Figure 6) was up-regulated from 26 hpi to 72 hpi and 144 hpi (R = 10.2, R = 33.7 and R = 47.3, respectively) comparatively to the control (R = 2, R = 1.3 and R = 1.2, respectively) (p < 0.05). In family A, the expression level of MyD88 increased significantly (p < 0.05) at 12 hpi and 26 hpi in infected oysters (R = 28.4 and R = 122.2, respectively) comparatively to its level of expression in negative control oysters (R = 1.2 at 12 hpi and R = 0.9 at 26 hpi) (Figure 6). In family P, MyD88 transcript levels decreased at 12 hpi (R = 0.2) and subsequently increased at 26 hpi (R = 20.4 fold) as compared with their level in negative control oysters (p < 0.05). However, MyD88 transcript levels were similar in infected and negative control oysters at 72 and 144 hpi. For the IAP oyster gene, transcript levels increased significantly (p < 0.05) at 26 hpi in infected oysters (R = 25.5) versus controls (R = 2.1) of family A. In family P, this level was also up-regulated at 26 and 72 hpi in infected oysters (R = 11.9 and R = 5.2, respectively) versus control oysters (R = 1.5 and R = 1.2 respectively) (p < 0.05) (Figure 7). For IkB2 gene, the number of transcripts increased at 26 hpi in infected oysters in both A and P families (R = 6.4 and R = 3.7 respectively, p < 0.05) (Figure 6).For the Gly gene, no significant change was observed between infected and control oysters in family A. In family P, the Gly transcript level (Figure 7) was down-regulated at 8 hpi and 26 hpi (R = 1.7 and R = 0.1 respectively) in infected oysters compared to controls (R = 1.2 and R = 2.5 respectively). Then Gly transcript levels were up-regulated at 144 hpi in infected oysters versus controls (R = 3.2 versus R = 1.3, p < 0.05).

Throughout the experiment, the relative expression of these genes did not follow a consistent pattern as they were alternatively up-regulated or down-regulated. This is particularly noticeable in the case of MyD88, IFI44 and Gly.

Using Spearman’s test, a rank correlation was determined between the expression of oyster genes and viral DNA amounts. Expression levels of MyD88 (Rs = 0.7), IkB2 (Rs = 0.43), IFI44 (Rs = 0.59) and IAP (Rs = 0.47) were positively correlated with viral DNA amount in family A. In family P, a positive correlation was observed only for IFI44 (Rs = 0.46) and IAP (Rs = 0.48) (Table 2).

**Table 2:** **Viral load and correlation with the levels of expression of four selected in family A and family P (Rs Spearman correlation)**

Oyster genes	Family A		Family P	
	Rs	***p value***	Rs	***p value***
MyD88	0.70	< 0.0001	0.23	0.09
IkB2	0.43	< 0.0001	0.10	0.46
IFI44	0.59	< 0.0001	0.46	< 0.0001
Gly	-0.18	0.26	-0.03	0.83
IAP	0.47	< 0.0001	0.48	< 0.0001

In order to compare oyster gene expression levels between families A and P, an Rf ratio was calculated (Rf = R of family A/R of family P). Our results show differences concerning the expression level of each gene during experimental challenge in controls in families A and P. Nevertheless, at 4 hpi down-regulation of five genes was reported in infected oysters from family A in comparison with family P. At 8 hpi, expression levels were similar in both families. At 12 hpi MyD88 were strongly up-regulated and Gly was down-regulated in infected animals belonging to family A compared to family P. Finally at 26 hpi, MyD88 and Gly were up-regulated in family A as compared with family P (Table 3).

**Table 3:** **Transcript ratios for each studied gene in families A and P (Rf = R family A/R family P) at different time points prior to the onset of mortality**

Genes	Control				Infected			
	4 h	8 h	12 h	26 h	4 h	8 h	12 h	26 h
MyD88	2.2	0.6	3.6	1.0	0.7	2.0	158.3	6.0
IkB2	0.9	0.7	1.3	1.8	0.7	1.0	1.5	1.7
IFI44	1.0	1.5	0.8	0.8	0.2	1.0	3.7	2.4
Gly	0.3	0.6	1.0	0.2	0.5	2.4	0.5	10.0
IAp	3.7	0.8	0.3	1.8	0.3	0.9	2.7	2.1

## Discussion

The objective of the present study was to compare the susceptibility of Pacific oyster families towards experimental infection by OsHV-1 and to further analyze the basis for contrasted susceptibility in two particular families. In this study, the injection protocol was selected in order to obtain a synchronous infection in all animals at the same time. In our experiments, survival at 120 hours post injection (hpi) was highly variable among the 16 tested families and remained diverse at 144 hpi between families A and P. This result suggests great variability of susceptibility to the viral infection in experimental conditions [22]. Sauvage et al. [10] previously suggested that a significant genetic component exists for susceptibility to the viral infection.

OsHV-1 DNA amounts increased sharply until 26 hpi in family A and to a lesser extent in family P until 72 hpi. The amount of detected OsHV-1 DNA decreased at 144 hpi in family P. The peak of viral DNA copy number preceded the peak of mortality for both families. These results suggest that the virus replicated better in family A, which was affected by the highest mortality rates. In family P, mortality rates remained low but OsHV-1 was able to replicate. Notwithstanding the development of an active infection some individuals belonging to this family appeared to recover from the infection. Conversely, Verrier et al. [23] showed that resistant cell lines obtained from rhabdovirus-infected fish clones did not support virus production whereas strong virus production took place in a highly susceptible cell line. On the contrary, Purcell et al. [24] reported results that are consistent with those observed in the present study, as resistant rainbow trout families showed lower Infectious hematopoietic necrosis virus (IHNV) replication, particularly at very early stages of infection. Others authors investigated the genetic basis underlying contrasted susceptibility to Marek’s disease virus (MDV) in chicken through experimental trials [25, 26].

Heatmap approach was selected to compare the global viral gene expression profiles for the 39 selected OsHV-1 genes in infected Pacific oysters from families A and P.

Although mortality rates remained very low for family P, virus gene transcripts were detected. Expression levels were clearly different between family A and family P. Cluster studies showed that viral ORFs were over-expressed in family A as compared with family P during the course of time. However, most of the analyzed viral genes (82%) had a similar expression profile in both families. Three different expression profiles were observed in both families: low, moderate and high expression. Among the latter, viral IAP genes (ORF 42, ORF 87 and ORF 99) were identified, suggesting that OsHV-1 may actively manipulate host apoptosis in order to multiply itself. Interestingly, in oysters from the family demonstrating the highest susceptibility to the viral infection (family A) all 39 ORFs were up expressed at 26 hpi in comparison to the less susceptible one (family P). As for virus DNA detection, expression of gene transcripts confirmed that OsHV-1 was able to replicate in oysters from family P to a lesser extent than in oysters from family A. Although virus infection effectively developed in Pacific oysters from family P, most animals appeared to recover from the infection. Moreover, a decrease in the amount of virus transcripts until they became undetectable at 144 hpi was recorded in family P. This result suggests that the virus may enter a persistence/latency phase as described in vertebrate herpesviruses. However, 70% of OsHV-1 genes encode putative proteins with unknown function, which makes the study of virus interactions and of the viral cycle particularly difficult. Other studies would be necessary to further unravel the development of the cycle of OsHV-1, including functional genomics analysis.

Our data also shows high variability of virus gene expression in both oyster families. This could be explained by the involvement of more than one OsHV-1 genotype in the infectious process. However, partial sequencing and genotyping of six viral microsatellite loci indicated the presence of a unique OsHV-1 genotype (μVar), in both families after the onset of viral infection (data not shown). The observed variability may therefore be interpreted as the presence in each oyster family of individuals with different degrees of susceptibility to the same OsHV-1 genotype.

These results confirm a genetic basis for susceptibility to OsHV-1 infection in the Pacific oyster [10, 11]. Moreover, they suggest that oysters belonging to family P, the less susceptible one, could be able to circumvent infection by OsHV-1 after experimental inoculation of the virus challenge. In this family, a phase of active virus replication took place, as demonstrated by the initial increase of viral DNA and RNA. Ensuing this phase, virus DNA and RNA amounts decreased rapidly suggesting that the host immune defense had been activated and that it might be effective to limit virus multiplication [27, 28]. To explore this hypothesis, the expression of immune related genes [20, 29] was also monitored by real time PCR.

The expression levels of five host genes (Myeloid differentiation 88 (MyD88), Interferon induced protein 44 (IFI44), Glypican (Gly), Inhibitor of nuclear factor kappaB kinase beta (IkB2), Inhibitor of Apoptosis (IAP)) were thus analyzed in families A and P. In our study, transcript ratios shown small differences (Rf > 1) in control oysters suggesting a family effect on the gene expression. Nevertheless, this ratio was highly different in infected oysters, especially at 12 and 26 hpi for MyD88. It was previously shown that the expression of three of these genes (MyD88, IFI44 and Gly) in *C. gigas* haemocytes is modulated after a contact with OsHV-1 [20]. In the case of IkB2, decreased abundance of its transcripts was associated with a certain level of resistance to summer mortality [13]. Finally, an inhibitor of apoptosis (IAP) was selected for this study due to the fact that it could play a pivotal role in the Pacific oyster immune defense [30].

MyD88 transcripts were up-regulated at 26 hpi in infected Pacific oysters from both families, although up-regulation was higher in family A (Rf = 6). In this family, the expression level of MyD88 was positively correlated with viral DNA amounts in family A (Rs = 0.70, p < 0.00001) whereas no significant correlation was detected in family P (p > 0.05). MyD88 is a universal adapter for the TLR/IL-1R family (TIR). It has been described in many species including humans [31], fish [32], drosophila [33], and molluscs [34]. Several studies demonstrated that MyD88-deficient mice were susceptible to HSV-1 infection [35, 36]. The signaling pathway via MyD88 is initiated to activate nuclear factor-kappa B (NF-kB), c-Jun NH2 terminal kinase (Jnk) and mitogen-activated proteins kinase (MAPKs) [37]. IkB2 transcripts showed significant up-regulation only at 26 hpi in both families and expression levels were almost identical in both families (Rf = 1.7). Nevertheless, the positive correlation of the expression level of IkB2 and viral DNA amounts was only observed in family A (Rs = 0.43, p < 0.0001). IkB2 is a member of NF-kappaB signal pathway and plays an important role in regulating the innate immune response of invertebrates. Zhang et al. [38] described IkB2 mRNA up-regulation in hemocytes at different time-points (2 and 24 hpi) after contact with *Vibrio alginolyticus* in pearl oyster. Over-expression of both MyD88 and IKB2 genes suggests a crucial role of the NF-kappaB signal pathway in virus recognition and cell activation [39]. Some viruses, such as the African swine fever virus (ASFV) [40], HIV [41, 42] or EBV [43] have evolved strategies to interfere with NF-kB activation in order to evade the immune response. However, viruses can also activate NF-kB to block apoptosis and prolong survival of the host cell in order to gain time for replication and increase the production of viral progeny [44].

Although MyD88 is reported as a key element in the activation of immunity, over-expression of this gene appeared higher in Pacific oysters belonging to family A, which is more susceptible to OsHV-1 infection. In this context, the over expression of MyD88 could be interpreted more as a marker of infectious processes and viral replication than that of an effective antiviral response. Gagnaire et al. [45] previously suggested that gene over-expression in Pacific oysters could lead to tissue injury and thus result in higher mortality rates. Defense mechanisms may play a key role in pathogenesis as they induce cell and tissue damages [46, 47].

Here, IFI44 was highly up-regulated in both families and continued to increase in family P at 72 hpi. The detection of increasing levels of IFI44 transcripts in oysters from this family between 72 hpi and 144 hpi was thus concomitant with the detection of decreasing amounts of OsHV-1 DNA and RNA by real-time PCR. Nevertheless, the analysis of data collected throughout the entire experiment reveals an overall positive correlation between the expression of this gene and viral DNA amounts in both families (Rs = 0.46, p < 0.0001). This result suggests that IFI44 may be a key element for effective antiviral defense against infection by OsHV-1 infection as it provides Pacific oysters with the ability to circumvent the virus proliferation. IFI44 gene is a member of the IFNs (IFNα/β) inducible gene family and may function as a mediator of antiviral activity against hepatitis C or D virus infections through interferons [48, 49]. However, the role of IFI44 in antiviral immune response remains unclear.

The glypican transcript level decreased significantly 8 hpi in family P whereas no change was observed in family A as shown by the Rf value. Glypicans belong to a family of heparan sulfate proteoglycans that are linked to the cell surface by a glycosylphosphatidylinositol (GPI) anchor [49]. Heparan sulfate proteoglycans may be used by different viruses including herpesviruses as cellular receptors [50]. Binding of a viral protein to heparan sulfate is the first step in a cascade of interactions between viruses and cells that is required for viral entry into the cells and the initiation of infection. CHO cells treated with heparinases that prevent heparan sulfate biosynthesis have reduced capacity to bind HSV-1 and are at least partially resistant to HSV-1 infection [51]. Therefore, our results suggests that reduced glypican levels in family P may obstruct or hinder to some extent OsHV-1 entry in host cells and contribute to this family decreased susceptibility to viral infection.

IAP cellular transcripts were respectively up-regulated in families A and P between 12 hpi and 26 hpi and between 26 hpi and 72 hpi. Our study showed a positive correlation between the level of IAP transcripts and the amounts of viral DNA in both families (Rs = 0.47, p < 0.0001 for family A and Rs = 0.48, p < 0.0001 for family P). IAP proteins are conserved throughout animal evolution and can block apoptosis. Apoptosis is one of the major mechanisms of anti-viral response [52]. Over-expression of IAP could be a reaction to the apoptotic process induced by OsHV-1 infection. Nevertheless, some pathogens enter a cell and inhibit apoptosis to increase their life span [53]. All the members of the gamma-herpesvirus family encode genes that are able to inhibit apoptosis including, one or two Bcl-2 homologues [54]. Moreover, studies have shown that HSV-1 require gene Us5 to protect itself from apoptosis induced by certain stimuli [55]. Finally, the contribution of apoptosis to the pathogenesis of West Nile Virus (WNV) encephalitis has been demonstrated by several studies [56, 57].

## Conclusion

This work is the first step towards elucidating the interactions between OsHV-1 and its host, the Pacific oyster (*C. gigas*). It also represents the first study of viral gene expression in oyster families with contrasted susceptibility to virus infection. In two particular families, contrasted susceptibility was associated with significantly different amounts of virus DNA and RNA, which suggests that susceptibility of Pacific oysters to OsHV-1 may be affected by genetic variations. Previous studies support the hypothesis of a genetic basis underlying resistance to OsHV-1 infection in the Pacific oyster. In addition, the observed differences in terms of susceptibility to infection by OsHV-1 were associated with differences in host gene expression profiles. Finally, the present study is also the first to show positive correlations between viral DNA amounts and the expression of selected oyster genes. These results suggest that the up-regulation of immune related genes may prevent virus replication and open new questions regarding the persistence and/or latency of the virus within the host. Genes of interest could be further evaluated in oyster breeding programs to determine if they confer genetic resistance to OsHV-1. Such an integrative strategy could be applied to other infectious diseases.

## Methods

### Animals

Wild oysters *Crassostrea gigas* were randomly sampled from an oyster reef in the Marennes-Oléron Bay in January 2011. The broodstock was acclimated gradually to 21°C over an 8-day period, in one 120 L tank, with a flow of 400 L/h during two months. In order to favor the gametogenesis, a cultured phytoplankton diet was added to the seawater with a continuous flow of 5 L/h (1 million cells/mL) during this period (10% *Isochrysis galbana*, 10% *Chaetoceros pumilum*, 10% *Tetraselmis suecica*, 10% *Pavlova lutheri* and 70% *Skeletonema costatum*). Sixteen bi-parental families, respectively named A to P were produced in the present study in March 2011 at the Ifremer hatchery in La Tremblade (Charente Maritime, France) during the course of the Bivalife EU funded project (FP7, 2011–2014). For each parent, spermatozoids or oocytes were individually collected by stripping the gonad of males and females respectively, and each crossing was performed following a previously reported protocol [58], and reared as described in Ernande et al. [22].

No specific permits are required at national and European levels for the described experimental study. The Pacific oyster *C. gigas* is not an endangered or a protected species.

### Defining OsHV-1 susceptibility of 16 Pacific oyster families

Experimental infections were carried out using the 16 produced families (9 months old, 3 cm). Thirty spat of each family were “anaesthetized” during 4 h in a solution containing magnesium chloride (MgCl_2_, 50 g/L) in a mixture of seawater (1 v)/ distilled water (4 v) [59]. One hundred μL of an OsHV-1 (μVar, [21]) suspension adjusted at 10^3^ copies of viral DNA/μL were injected into the adductor muscle of each individual using a 1 mL syringe [59]. Pacific oysters were then placed in 3 tanks (10 oysters per tank) containing 5 L of filtered seawater (1 μm) at 22°C. Similarly, 30 spat from each family were injected with 100 μL of artificial seawater as a control. Mortality was checked every day. Two similar assays were conducted in October 2011.

### Experimental infection of two oyster families presenting contrasted OsHV-1 susceptibility

A third trial was carried out. One hundred and sixty oysters (10 month old, 3 cm) from families A and P were “anesthetized” during 4 h as previously described. One hundred μL of artificial seawater or a viral suspension at 10^3^ copies of viral DNA/μL (μVar) were injected into the adductor muscle of 80 oysters for each family as already reported [59]. Oysters were then distributed in 4 tanks per condition (seawater vs viral suspension) and per family (20 animals per tank). One tank per condition was dedicated to record survival and the three others were dedicated for the sampling.

Four live oysters per tank, condition and family were collected 0, 4, 8, 12 and 26 hours post-injection (hpi) for both families, as well as 72 hpi and 144 hpi for family P. Two pieces of mantle were sampled from each individual. The first piece (50 to 100 mg) was disposed in a tube containing 1 mL of TRIZOL® Reagent™ (Ambion®) and frozen at -80°C for RNA extraction. The second piece was directly frozen at -20°C in a tube for DNA extraction.

### Total DNA or RNA extraction and cDNA synthesis

DNA extraction was performed with QiAamp tissue mini kit® (QIAgen) according to the manufacturer’s protocol. Elution was performed in 100 μL of AE buffer provided in the kit.

Total RNA was extracted using TRIZOL® Reagent™ (Ambion®) according to the manufacturer’s recommendations. Total RNA was treated with Turbo™ DNAse (Ambion®) to remove genomic DNA. The RNA quality and quantity were determined using NanoDrop 2000 (Thermo Scientific) and Bioanalyser 2100 (Agilent). First-strand cDNA synthesis was carried out using the SuperScript® III First-Strand Synthesis System (Invitrogen) using 8000 ng of RNA treated. A No RT was performed after each DNAse treatment using real time PCR in order to control absence of oyster and/or virus genomic DNA using EF primers (Table 4) and C9/C10 primers [60] respectively.

**Table 4:** **List of oyster genes targeted by real time PCR**

GenBank	EST name	Abbreviation	Forward primer	Efficiency (%)	Primer reference
			Reverse primer		
DQ530619	Myeloid differentiation factor 88	MyD88	CGTGCCATGGACGGATAACAACG	97.5	[20]
			GGCCCAGCAGTACCTCTGTGGAATC		
AM856743	NF-Kappa-B-inhibitor cactus	IkB2	CAGCATTCACTGACGACGAT	104	[14]
			TCTGCCTCAGTTTGTCGTTG		
FJ440108	Interferon Induced Protein 44	IFI44	TGGTGGACTATGGACCGGACAGTG	93.8	
			GGTAGACAGTGGTGAGGCTGTGCT		
EU678311	Glypican	Gly	AACTACTGCCTCAACACCATGAA	102.1	
			TAGGTGTCACCAACAGAACCAC		
JH818926	Baculoviral IAP repeat-containing protein 2	IAP	CCCGAAAACGTAACCTCAGA	103	Barbosa-Solomieu et al., pers com
			TTTCGTTTGCTGCTCATTTG		
AB122066	Elongation Factor	EF	AGTCACCAAGGCTGCACAGAAAG	98.8	
			TCCGACGTATTTCTTTGCGATGT		

### OsHV-1 DNA quantification and gene expression by real-time PCR

#### OsHV-1 DNA quantification

OsHV-1 DNA quantification was carried out in the third trial using a real-time PCR protocol [61]. Real-time PCR was performed in duplicate using a Mx3000 Thermocycler sequence detector (Agilent). Amplification reactions were performed in a total volume of 20 μL. Each well contained 5 μL of genomic DNA (5 ng/μL), 10 μL of Brillant III Ultra-Fast SYBR® Green Master Mix (Agilent), 2 μL of each primer (3 μM) and 1 μL of distilled water. Real time PCR cycling conditions were as follow: 3 min at 95°C, followed by 40 cycles of amplification at 95°C for 5 s, 60°C for 20 s. Melting curves were also plotted (55-95°C) in order to ensure that a single PCR product was amplified for each set of primers. Negative controls (without DNA) were included.

#### Viral gene expression

Real-time quantitative RT PCR was used to study the expression of 39 viral genes using the previously described protocol with 5 μL of cDNA dilution (1/30) instead of genomic DNA [18]. Normalized relative viral gene expression levels were calculated for each individual with formula: F = log_10_[(E + 1)^40-Ct^/N] adapted from De Decker et al. [62], where E is efficiency of each ORFs [18], Ct (threshold cycle) corresponds to the PCR cycle number, N is the maximal number (10^7^) of viral DNA copies/ng of total DNA detected minus number of viral DNA copies/ng of total DNA determinate by absolute real time PCR for each individual and Ct = 40 is arbitrarily considered to correspond to 'no Ct’ obtained by real time PCR.

#### Host gene expression

Moreover, the relative expression of five genes from *Crassostrea gigas* spat were studied during the third OsHV-1 experimental infection at 4, 8, 12, 26 hpi for both families and 72 and 144 hpi for family P. The relative quantification value (ratio R) was calculated using the method described by Pfaffl [63]: R = [(E_target_)^ΔCT target(control-sample)^]/[(E_EFα-1_)^ΔCT EFα-1 (control-sample)^]. The efficiency of each primer pair was determined by constructing a standard curve from serial dilutions (Table 4). These 5 genes from Pacific oyster were (i) myeloid differentiation factor 88 (MyD88), (ii) NF kappa-B inhibitor cactus (IkB2), (iii) interferon-induced protein 44 (IFI44), (iv) glypican (Gly), (v) Baculoviral apoptosis inhibitor repeat-containing protein 2 (IAP) genes (Table 4). Host gene expression was normalized to the elongation factor I (EF), as no significant differences of Ct values were observed for this housekeeping gene between several conditions during the course time (CV = 3.2%, Kruskal-Wallis test Z = 0.19, p > 0.05). Nevertheless, the variance analysis of EF Ct value demonstrated a significant difference were between families (Kruskal-Wallis test, Z = 2.19, p < 0.05). The calibrator used for the experiment consisted of individuals sampled at time 0 hpi from each family.

### Data analysis

Kaplan-Meier survival curves and the Wilcoxon test were used to characterize and compare survival between oyster families.

Cts were calculated with the Stratagene Mxpro software 4.0. A heatmap was generated in order to represent the viral gene expression using the 'pheatmap’ package in R. Clustering of viral genes was performed using the euclidean method. Results for relative expression of oyster genes were expressed as boxplot. Kruskal-Wallis and Dunn’s tests were used to analyze viral clusters, and a Mann–Whitney test was used to analyze oyster gene expression.

Correlations between viral load and oyster gene expression were tested with the Spearman’s nonparametric rho (Rs). All statistical computations were performed with the XLSTAT software (version 2013), and results were declared statistically significant at the two-sided 5% (*i.e.*, P < 0.05). The Rf ratio was calculated using the expression level (R) in family A/ the expression level (R) in family P in order to compare the oyster gene expression between both families.
